# Segmented intravaginal ring for the combination delivery of hydroxychloroquine and anti-CCR5 siRNA nanoparticles as a potential strategy for preventing HIV infection

**DOI:** 10.1007/s13346-021-00983-w

**Published:** 2021-04-17

**Authors:** Yannick L. Traore, Yufei Chen, Fernanda Padilla, Emmanuel A. Ho

**Affiliations:** 1 Laboratory for Drug Delivery and Biomaterials, School of Pharmacy, University of Waterloo, Kitchener, Canada; 2 Waterloo Institute for Nanotechnology, Waterloo, Canada; 3 College of Pharmacy, University of Manitoba, Winnipeg, Canada

**Keywords:** HIV, Intravaginal ring, pH-responsive polymer, Anti-CCR5 siRNA, Solid lipid nanoparticles

## Abstract

**Abstract:**

Vaginal drug delivery has been shown to be a promising strategy for the prevention of sexually transmitted infections. Therapy delivered at the site of infection has many advantages including improved therapeutic efficacy, reduction in systemic toxicity, and reduced potential for development of drug resistance. We developed a “smart” combination intravaginal ring (IVR) that will (1) provide continuous release of hydroxychloroquine (HCQ) to induce T cell immune quiescence as the first-line of defense and (2) release nanoparticles containing anti-CCR5 siRNA only during sexual intercourse when triggered by the presence of seminal fluid as the second-line of defense. The IVR was capable of releasing HCQ over 25 days with a mean daily release of 31.17 ± 3.06 µg/mL. In the presence of vaginal fluid simulant plus seminal fluid simulant, over 12 × more nanoparticles (5.12 ± 0.9 mg) were released over a 4-h period in comparison to IVR segments that were incubated in the presence of vaginal fluid simulant alone (0.42 ± 0.19 mg). Anti-CCR5 siRNA nanoparticles were able to knockdown 83 ± 5.1% of CCR5 gene expression in vitro in the CD4^+^ T cell line Sup-T1. The IVR system also demonstrated to be non-cytotoxic to VK2/E6E7 vaginal epithelial cells.

**Graphical abstract:**

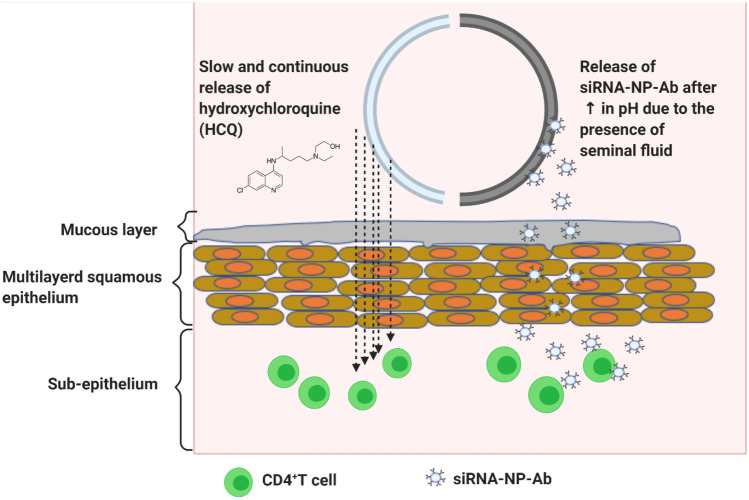

## Introduction

Despite the efforts that have been made to date, HIV is still one of the leading causes of death in developing countries. More than 38 million people globally are living with HIV at the end of 2019 with women being more vulnerable due to biological and social-cultural factors. Reasons include the larger surface area of the female genital tract (FGT) in comparison to the male genital tract and the fact that after sexual intercourse, semen remains within the FGT for prolonged periods, increasing exposure to HIV [1–3]. Condoms remain as the best method for preventing HIV infection during heterosexual intercourse, but unfortunately, in certain developing countries, women are not able to negotiate condom usage with their partners [4]. Furthermore, due to the high rate of mutation by HIV, most conventional drugs are not effective due to the development of drug resistance. To reduce the development of multidrug resistance, therapy involving different classes of antiretrovirals is necessary [5]. However, this multidrug therapy can pose as a burden for patients especially when it must be taken daily. As one example, pre-exposure prophylaxis (PrEP) consisting of tenofovir and emtricitabine, both reverse transcriptase inhibitors and marketed as Truvada®, demonstrated high efficacy against HIV-1 [6]. Interestingly, a study has reported that low patient compliance to Truvada may result in the development of antiviral drug resistance [7, 8]. Hence, the necessity to develop an easy and effective therapy against HIV that does not require daily drug administration is required. A great strategy is to develop microbicides (self-administrated topical PrEP) that do not require much effort from the user [9]. An ideal microbicide would be one that is long-acting and is capable of delivering high dose of drug to the target site demonstrating therapeutic efficacy and low toxicity. A common challenge with microbicide development as observed in clinical trials is low patient adherence [10]. In addition, some microbicides have also elicited inflammation within the FGT resulting in increased HIV infection rates [11–13]. Different microbicides have been developed in the past several decades including vaginal gels, creams, suppositories, bio-adhesives, and intravaginal ring [9, 14]. Intravaginal rings (IVRs) are an excellent alternative that can provide controlled and sustained release of an active ingredient within the FGT for an extended period [15]. IVRs have high acceptability amongst users and can be designed to deliver different active ingredients using the same ring [16, 17]. Furthermore, in a recent clinical trial, it was reported that placebo IVRs did not significantly induce an inflammatory response within the FGT of women suggesting that IVRs are safe for long-term use, e.g., 28 days [18]. In this study, we developed a dual-combination IVR system designed to prevent HIV infection by incorporating two distinct approaches. The first strategy will be to induce an immune quiescent state using hydroxychloroquine (HCQ) to reduce the number of HIV target cells within the FGT. Secondly, our IVR system will utilize gene therapy to knockdown the expression of CCR5 using small interfering RNA (siRNA) to reduce HIV binding to target cells.

It has been shown that high CD4^+^ T cell immune activation strongly correlates with HIV infection [19]. CD4^+^CCR5^+^ immune cells are the primary target cells for HIV. While activated, there will be an increase expression of cell surface markers such as HLA-DR, CD69, and CD38 resulting in an increase in the susceptibility for HIV infection [20]. Studies have shown that HIV-exposed seronegative women exhibit a unique immune phenotype called immune quiescence, whereby they exhibit a lower baseline of T cell immune activation when compared to people that are at a greater risk of contracting HIV [20, 21]. HCQ, a widely available FDA-approved immunomodulatory drug used for the treatment of malaria, systemic lupus erythematosus, and rheumatoid arthritis, has been shown to reduce HIV viral loads and induce T cell immune quiescence [22–24]. By using an IVR to deliver HCQ in a sustained and controlled manner at the site of infection e.g. FGT, it will greatly reduce viral infection during sexual intercourse. The immunomodulatory properties of HCQ are result of its ability to increase the pH of lysosomes within T cells, interfering with the association of the invariant chain of the major histocompatibility complex class II molecule leading to their reduced ability to process antigens [25, 26].

The second strategy will be to use siRNA directed against the CCR5 receptor expressed at the surface of T cells. HIV will bind to CD4 and CCR5 receptors at the surface of T cells to become internalized. Studies have shown that knocking down the CCR5 gene will prevent HIV-1 infection. For example, the first patient to be completely “cured” from HIV received a bone marrow transplant from a donor who had a rare stem cell mutation that prevented the expression of CCR5 on T cells. Three months after the transplant, the patient was completely cured of HIV [27]. Recently, a second case of another patient following the same treatment using CCR5 knockout stem cell transplant received from a donor was also cured of HIV [28]. Since it will be challenging to find sufficient donors for all HIV patients, using gene therapy to knockdown the CCR5 expression is a promising approach. In our study, we plan to deliver siRNA to target the CCR5 gene as a strategy for reducing HIV infection [29, 30]. siRNA being negatively charged cannot cross the cell membrane, and as a result, requires a carrier system such as solid lipid nanoparticles (SLN) to deliver siRNA into T cells. SLNs were used due to its demonstrated biocompatibility and simplicity in terms of customization. For active targeted delivery to immune cells, SLNs were functionalized with anti-CD4 antibody. The attached CD4 antibody will facilitate binding of SLNs to the CD4 receptor on the surface of T cells resulting in improved internalization.

Our study aims to develop and characterize a segmented combination IVR delivery system consisting of one-half of the IVR loaded with HCQ and the second-half of the IVR to be coated with a pH-responsive film loaded with siRNA-encapsulated SLNs. It is expected that at normal vaginal pH (3.5–4.3), there will be no release of siRNA-loaded nanoparticles. During heterosexual intercourse, the vaginal pH becomes elevated (> 6.2) due to the presence of seminal fluid [31]. As a result, it is expected that at pH > 6.2, the IVR will provide rapid release of siRNA-nanoparticles, while HCQ will be released continuously over time at both acidic and neutral pH to prevent immune activation of T cells. This novel dual combination microbicide has the potential to improve patient compliance and reduce HIV infection.

## Materials and methods

### Materials

Hydrophilic thermoplastic polyurethane (PU) (Tecophilic™ HP-60D-35) was purchased from Lubrizol Advanced Materials (Cleveland, OH, USA). Eudragit L100 (methacrylic acid-methyl acrylate copolymer; anionic pH-sensitive polymer) was kindly donated by Evonik Industries (Essen, Germany). Hydroxypropyl methylcellulose (HPMC) K100M was donated by Dow Chemical Company (New Milford, CT, USA). Polyethylene glycol 400, NF (PEG-8) was acquired from Medisca (Saint-Laurent, QC, Canada). HCQ was purchased from Thermo Fisher Scientific (Burlington, ON, Canada). CellTiter 96® AQueous One Solution Cell Proliferation Assay (MTS) was purchased from Promega (Madison, USA). Glyceryl monostearate (molecular weight 358.56) was purchased from Sigma-Aldrich (Ontario, Canada). L-α-phosphatidylcholine (Soy-95%) (molecular weight 770.123 g/mol) was purchased from Avanti Polar Lipids (AL, USA). Polyvinyl alcohol (PVA; 31 − 50 kDa) and polyethyleneimine (PEI; branched, MW 25 K) were obtained from Sigma-Aldrich (Ontario, Canada). Tris–EDTA was purchased from ThermoFisher Scientific (Ontario, Canada). 2-(N-morpholino) ethanesulfonic acid (MES) was purchased from Sigma-Aldrich (Ontario, Canada). 1-Ethyl-3-(3-dimethylaminopropyl) carbodiimide (EDC; 200 mg/mL) N-hydroxysuccinimide (NHS; 275 mg/mL) were purchased from G-Biosciences (Missouri, USA). Anti-Human CCR5 siRNA (sense: 5′-GUUCAGAAACUACCUCUUAdTdT-3′, antisense: 3′-dTdTCAAGUVUUUGAUGGAGAAU-5′) was purchased from Dharmacon (ON, Canada). Human CCR5 primers (Forward: 5′-TTCATCATCCTCCTGACAATCG-3′; Reverse: 5′-GCCACCACCCAAGTGATCAC-3′) and human GAPDH primer (Forward: 5′-AAGAAGGTGGTGAAGCAGGCG-3′; Reverse: 5′-AGACAACCTGGTCCTCAGTGTAGC-3′) were purchased from Thermo Fisher (ON, Canada). E.Z.N.A.® RNA isolation kit was purchased from Sigma Aldrich (ON, Canada). PerfeCTa SYBR® Green SuperMix and qScript™ cDNA were purchased from Quanta (ON, Canada). Anti-CD4 antibody was purchased from Abcam (ON, Canada).

### Method

#### IVR fabrication

**Fig. 1:**
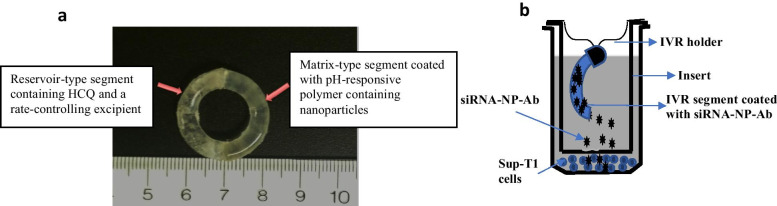
**a** The picture depicts a segmented intravaginal ring (IVR) formed by combining a reservoir-type segment with a matrix-type segment. The size of the IVR (25 mm × 5 mm) is designed for implantation into the female genital tract of non-human primates. IVR segments were fabricated using hot-melt injection molding of HP-60-D-35 polyurethane pellets and joined by butt-welding. **b** Schematic representation of the IVR segment in a cell culture insert incubated with Sup-T1 cells in a 24-well plate

“Reservoir-type” and “matrix-type” IVR segments were fabricated from medical grade PU (HP-60-D-35) pellets using hot-melt injection molding (Medium Machinery, LLC, Woodbridge, VA, USA). Briefly, the pellets were dried overnight at 80 °C and loaded into a hot-melt injection molding. The PU was then melted inside the injection molder and injected into a custom-fabricated, pre-heated aluminum mold for the reservoir-type or matrix-type segment. The mold produced IVR segments with a 25-mm outer diameter and 5-mm cross-sectional diameter for the matrix type IVR. The reservoir type IVR had a wall thickness of 0.75 mm (Fig. 1a), 25-mm outer diameter, and 5-mm cross-sectional diameter. The reservoir-type segment containing HCQ in the lumen was joined to the matrix segment using a butt-welding kit.

#### HCQ loading in reservoir-type IVR

HCQ was mixed with HPMC K100M to create a semisolid as described previously [32]. Briefly, 160 mg of HCQ was added to 1.28 mL of distilled water and mixed using the two-syringe method. About 160 mg of HPMC K100M was added to the HCQ solution and mixed using the two-syringe method. The semisolid was passed through the syringes 80 times to ensure homogeneity. The mixture was loaded into a reservoir-type IVR segment with both ends sealed with resin caps pre-made using Smooth-Cast 300.

#### Nanoparticle fabrication

SLNs encapsulated with siRNA (siRNA-NP) were prepared using a double-emulsion solvent evaporation technique as previously described with slight modifications [33]. We added 50 µL of 0.372 mg/mL PEI to a solution containing 45 µL of Tris–EDTA buffer and 5 µL of 25 µg siRNA. The mixture was incubated at room temperature for 15 min and mixed periodically. This aqueous solution was added slowly to 1.4-mL solution of glyceryl monostearate and L-α-phosphatidylcholine dissolved in acetone and ethanol and sonicated for 30 s on ice using a probe sonicator. The double emulsion was formed by adding this mixture to 8 mL of 2% (w/v) PVA solution. The organic solvents were then evaporated, and the siRNA-NPs were washed, and the encapsulation efficiency was determined. Anti-CD4 antibody was conjugated to the siRNA-NP by activating the carboxyl group on the nanoparticle’s surface by the addition of EDC and NHS at pH 6 for 60 min. The antibody-conjugated nanoparticles (siRNA-NP-Ab) were washed with distilled water, lyophilized, and stored at − 20 °C until further analysis. Nanoparticle size and net surface charge were determined by light scattering using Zetasizer (Malvern).

#### pH-responsive coating preparation

An optimized film formulation is necessary to achieve a uniform coating on the surface of the IVR. The coating should be able to stick to the IVR matrix when dried and not crumble or peel off when the IVR is compressed. For these reasons, we first evaluated the film forming capacity of Eudragit L100 and optimized the formulation that will work best for our IVR segments. Briefly, different percentages of Eudragit were dissolved in various organic solvents and evaluated for its film forming properties. The solvents include isopropanol, dichloromethane, methanol, dichloromethane:ethanol (50:50 v/v), and methanol:water (50:50 v/v). The mixture of organic solvent and Eudragit L100 was stirred at room temperature for at least 3 h. After the complete dissolution of the polymer, PEG400 was added at different ratios (Table 2) and stirred for another 10 min. In order to choose the best film formulation for the IVR coating, we performed a disintegration study to determine the rate of film disintegration. Briefly, 15 mg of Eudragit L100 films containing PEG were incubated in PBS buffer (pH 7.2) for 20 min. Afterwards, the remaining film was removed, dried in an oven at 37 °C, and weighed. For the complete disintegration time, we monitored the film until there was complete dissolution with no visible particles in the buffer. Next, after deciding on the formulation that gave the best adherence and the fastest disintegration time, siRNA-NP-Ab was added to the formulation (3% Eudragit L100 plus 0.2 mL PEG 400), mixed and sonicated for 30 min without heat to obtain an aqueous dispersion and to remove air bubbles. A matrix IVR segment was coated with the mixture and dried at 40 °C for 15 min.

#### In vitro release studies from reservoir-type and matrix-type IVR segments

Release studies were performed using reservoir-type IVR segments (19.6 ± 0.7 mm) containing HCQ and K100M in the lumen as previously described [32] in vaginal fluid simulant (VFS; pH 4.2) following the recipe proposed by Owen et al. [34]. Briefly, IVR segments (19.6 ± 0.7 mm) were placed in 5 mL of VFS at 100 rpm speed and 37 °C. The amount of drug released was quantified using a reverse-phase HPLC method. Isocratic conditions were used with a Waters Nopak® C18 column (4 µm, 3.9 × 150 mm) on Shimadzu LC-2010A HPLC system. The mobile phase consisted of 58-mM sodium phosphate dibasic buffer containing 15 mM of heptanesulfonic acid, acetonitrile, and methanol at a volume ratio of 74:22:4, and the pH was adjusted to 3.1 with ortho-phosphoric acid. The flow rate was set at 1 mL/min, and the wavelength of the detector was set at 343 nm. The column was kept at room temperature, and the HCQ retention time was approximately 8.4 min. We aim to achieve a targeted HCQ release of at least 4.3 µg/mL which is the minimum concentration required to induce T cell immune quiescence [35].

Separately, the pH-responsive coated matrix-type IVR segments were placed in two different release buffers. The first release buffer was VFS (pH 4.2), and the second buffer was a seminal fluid simulant (SFS; pH 8.2) prepared using a modified recipe of Rastogi et al. [36]. SLN containing coumarin 6 (C6) was prepared to help track NP release from the IVR segments. Briefly, a set of segments coated with Eudragit L100 containing C6 -SLN were placed in 4 mL of VFS. The volume of ejaculated seminal fluid is between 0.1 and 11 mL [37, 38]. Ejaculation volume < 1 mL is considered hypospermia, and more than 6 mL is considered hyperspermia. In this study, we decided to evaluate the lowest normal ejaculation volume capable of changing the vaginal tract pH. Accordingly, another set of IVR segments were placed in 4 mL of VFS + 1 mL of SFS to simulate the presence of seminal fluid during sexual intercourse and placed inside a rotary shaker at 37 °C and set at 100 rpm. The data was analyzed using a fluorescent microplate reader (SpectraMax M5).

#### In vitro CCR5 gene knockdown study

In order to evaluate the efficacy of our SLN to deliver siRNA inside the cells and to know the minimum amount of siRNA needed to induce gene knockdown, we treated Sup-T1 cells in 96-well plate with only different concentrations of siRNA-NP-Ab. Briefly, SupT-1 cells were cultured in RPMI medium supplemented with 10% fetal bovine serum and 1% penicillin–streptomycin incubated at 37 °C with 5% CO_2_. The cells were seeded in 96-well plate at a density of 2 × 10^4^ cells/well. Each well was treated with 1.4 mg, 0.7 mg, or 0.35 mg of siRNA-NP-Ab. The cells were incubated for 48 h at 37 °C with 5% CO_2_. After incubation, the cells were washed with PBS and processed for mRNA extraction using the E.Z.N.A.® Total RNA Kit I. cDNA was transcribed using qScript™ cDNA SuperMix in a thermal cycler (Bio-Rad C1000™ Thermal Cycler). RT-PCR was performed using the PerfeCTa SYBR Green SuperMix with GAPDH as an endogenous control.

For the next study, Sup-T1 cells were seeded in a 24-well plate at a density of 0.8 × 10^5^ cells per well. An insert was placed into each well, and an IVR segment coated with a pH-responsive polymer containing siRNA-NP-Ab (sterilized using ultraviolet light for 1 h) was placed into the insert. The IVR segments were suspended above the insert using a custom designed holder made out of stainless-steel wire (Fig. 1b). Negative control had an insert containing an IVR segment coated with blank NPs and another well with nothing but cells. The plate was placed on a shaker inside an incubator set at 2 rpm to allow dissolution of the coating for 48 h at 37 °C with 5% CO_2_. After incubation, we proceeded to the mRNA extraction and gene analysis as described above.

#### In vitro cytotoxicity studies of IVR containing nanoparticles

In vitro cytotoxicity was evaluated using the CellTiter 96® Aqueous One Solution Cell Proliferation assay. Briefly, VK2/E6E7 vaginal epithelial cells were cultured in K-SFM containing 0.1 ng/mL recombinant human epidermal growth factor, 44.1 mg/L calcium, 0.05 mg/mL bovine pituitary extract, and 1% penicillin–streptomycin. The cells were incubated at 37 °C with 5% CO_2_. Separately, this medium was used to incubate a matrix IVR segment coated with a pH-responsive polymer. The segment in the medium was incubated at 37 °C for 24 h at 100 rpm in an orbital shaker. After almost reaching confluency, the cells were seeded onto 96-well plates at a density of 2.5 × 10^5^ cells per well. The cells were then incubated for another 24 h, and the medium in each well was replaced with the elution medium collected previously. 1 M acrylamide was used as positive control and plain medium was used as negative control. The cells were incubated back for another 24 h followed by the addition of 20 µL of CellTiter 96® Aqueous One Solution Cell Proliferation assay reagent and incubated for another 2 h. The optical density of the wells was read using a microplate reader (SpectraMax M5) at 490 nm, and data were normalized to the negative control value. Previously, we have shown that HCQ-loaded reservoir segments were non-cytotoxic [32].

## Results

### IVR fabrication

A photo of the matrix-type and reservoir-type IVR joined together are shown in Fig. 1a. A complete macaque-size IVR has a diameter of 25 mm, and a cross-sectional diameter of 5 mm. The reservoir-type IVR segment has a wall thickness of ~ 0.7 mm and a length of 19.6 ± 0.7 mm. The lumen of the reservoir was filled with HCQ mixed with HPMC. The matrix IVR was coated with the modified pH-responsive polymer containing siRNA-NP-Ab. Both segments were joined together via butt-welding by applying heat to partially melt both ends of the different segments.

### Nanoparticle fabrication

The SLN size, net surface charge, and drug encapsulation efficiency were evaluated. The mean size of siRNA-NP-Ab was around 265.3 ± 15.7 nm with a polydispersity index of around 0.2. The siRNA encapsulation efficiency was 79.63 ± 7.3%, and the net surface charge was around − 26 ± 3.4 mV. Drug-free SLN had an average size of 210.5 ± 20.8 nm with a polydispersity index of 0.2.

### pH-responsive coating preparation

It is important to determine whether or not the selected solvent was capable of dissolving Eudragit L100. Table 1 lists the different solvents that were evaluated for their ability to dissolve Eudragit L100 and their film forming properties. Dichloromethane and dichloromethane:ethanol (50:50 v/v) were able to dissolve Eudragit L100 completely, but they also partially dissolved the PU HP-60D-35. Isopropanol was able to dissolve Eudragit L100 and did not have any effects on the PU. Table 2 summarizes the effects of varying amounts of Eudragit L100 and PEG 400 dissolved in isopropanol on film formation properties. The usage of low amounts of Eudragit L100 (3%) with low amounts of PEG 400 (0.1 mL) resulted in the formation of fragile and easy to break films. Higher amounts of PEG 400 (0.5 mL) hindered the film forming capacity of Eudragit L100 resulting in gel-like mixture. As a result, 3% Eudragit L100 dissolved in isopropanol supplemented with 0.2 mL of PEG 400 was used for all downstream studies since it produced the most uniform film with the fastest disintegration time. We observed rapid disintegration within the first 20 min (42%) followed by a reduced rate of disintegration for the next 2.5 h. Complete film dissolution was achieved in 3 h (Table 3). Only the formulations capable of forming a uniform and flexible film were considered in Table 3 for the disintegration study. The use of higher amounts of Eudragit L100 (> 3%) resulted in films with longer disintegration times (> 5 h).

**Table 1:** Eudragit L100 properties in different organic solvents and its impact on polyurethane

	Percentage of Eudragit L100			Film forming properties	Effects on polyurethane
Solvents	1%	3%	5%		
Methanol:Water (50:50 v/v)	Insoluble	Insoluble	Insoluble	No	None
Methanol	Partially soluble	Insoluble	Insoluble	No	None
Dichloromethane	Soluble	Soluble	Soluble	Yes	Dissolved
Dichloromethane:Ethanol (50:50 v/v)	Soluble	Soluble	Soluble	Yes	Partially dissolved
Isopropanol	Soluble	Soluble	Soluble	Yes	None

**Table 2:** Impact of varying amounts of Eudragit L100 and PEG 400 dissolved in isopropanol on film formation

Film formation							
				PEG 400 (mL)			
Eudragit (%)	0		0.1	0.2	0.3	0.4	0.5
3	Rigid film	Rigid film		Flexible	Gel state	Gel state	Gel state
4	Rigid film	Rigid film		Rigid film	Flexible	Gel state	Gel state
5	Rigid film	Rigid film		Rigid film	Flexible	Gel state	Gel state
7	Rigid film	Rigid film		Rigid film	Rigid film	Flexible	Gel state

**Table 3:** Eudragit L100 film disintegration time

Eudragit/PEG	PBS pH 4.0	PBS pH 7.2
	Disintegration time	
3%/0.2 mL	None	< 3 h
4%/0.3 mL	None	> 5 h
5%/0.3 mL	None	> 5 h
7%/0.4 mL	None	> 6 h
7%/0.5 mL	None	> 6 h

### In vitro release

**Fig. 2:**
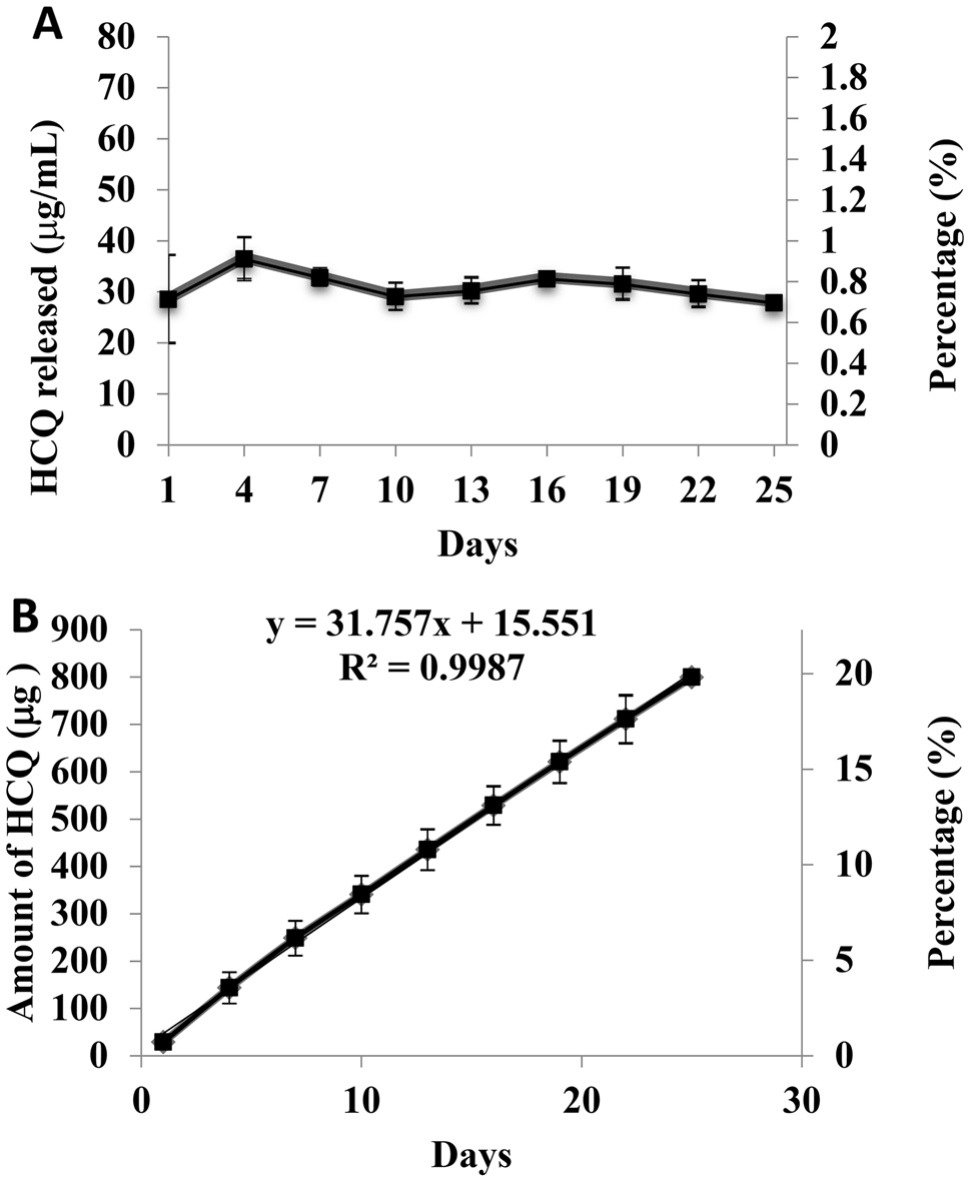
**a** Daily release of HCQ from HCQ/HPMC-loaded (ratio 1:1 wt/wt) IVR segments for 25 days. Release studies were performed in 5 mL of VFS (pH 4.2) and shaken on an orbital shaker at 37 °C, 100 rpm. Data represents mean ± S.D.; *N* = 4. **b** Cumulative release of HCQ from HCQ/HPMC-loaded (ratio 1:1 wt/wt) IVR segments for 25 days. Data represents mean ± S.D.; *N* = 4

**Fig. 3:**
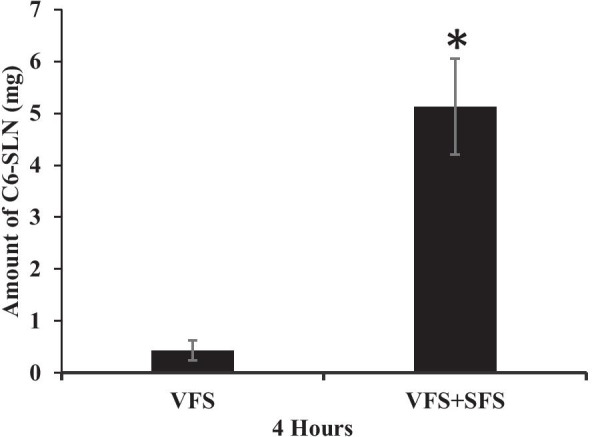
Release study of C6-SLN in VFS only and in VFS plus SFS to simulate the presence of seminal fluid during sexual intercourse. Data represents the mean ± SD (*n* = 4). *P < 0.05 versus VFS. C6 coumarin-6, VFS vaginal fluid simulant, SFS seminal fluid simulant

Release studies were performed on reservoir-type IVR segments containing HCQ/HPMC in VFS separately from the matrix-type IVR. We previously published a study evaluating the release of HCQ from HP-60D-35 IVR segments in sodium acetate buffer [15]. In this study, release was evaluated in VFS which more closely represents the physiological environment. HCQ released from the IVR segment was sustained over 25 days with a mean daily release of 31.17 ± 3.06 µg/mL following a near-zero release kinetic profile (*R*^2^ = 0.998) (Fig. 2). Over the period of 25 days, 19.81 ± 1.3% of the total amount loaded was released. The matrix-type IVR segment coated with the pH-responsive polymer containing C6-SLN was introduced in two different buffers for the release study. After 4 h, the segment placed in VFS released 0.42 ± 0.19 mg of C6-SLN, while the IVR segment that was incubated in VFS supplemented with SFS buffer released 12 × higher amounts of C6-SLN (5.12 ± 0.9 mg) (Fig. 3).

### Determination of the in vitro cytotoxicity of IVR containing SLNs

**Fig. 4:**
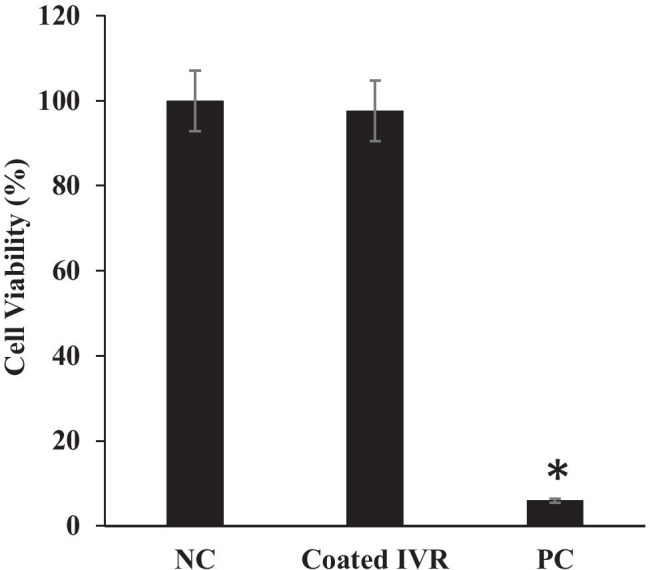
Cytotoxicity evaluation of IVR segments coated with Eudragit L100 on VK2/E6E7 vaginal epithelial cells. The IVR segment was coated with 15 mg of Eudragit L100 film. PC positive control (1 M acrylamide); NC negative control (cell media). Data represents mean ± S.D.; *N* = 4. *P < 0.05 versus NC

The safety profile of the reservoir-type IVR segments containing HCQ/HPMC was previously evaluated and published [32]. As a result, we focused only on the second half of the IVR in this study. As shown in Fig. 4, no significant cytotoxicity was observed in VK2/E6E7 cells incubated with matrix-type IVR segments coated with the SLN-loaded pH-responsive coating in comparison to the control.

### In vitro CCR5 downregulation study

**Fig. 5:**
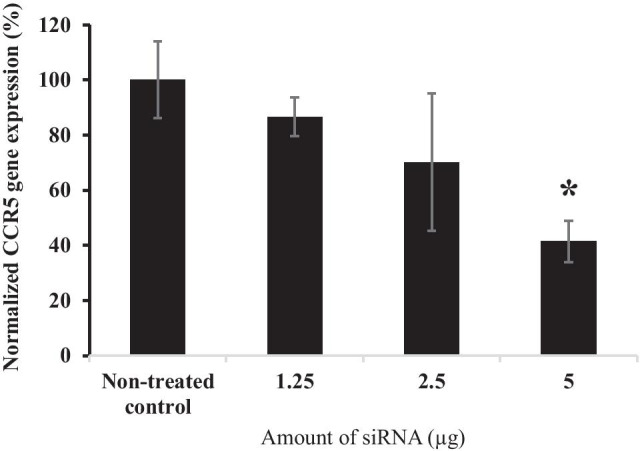
Evaluation of CCR5 gene expression using real-time PCR. The T cell line (Sup-T1) was treated with various concentrations of siRNA-NP-Ab for 48 h. Data represents mean ± S.D.; *N* = 3. *P < 0.05 versus non-treated control

**Fig. 6:**
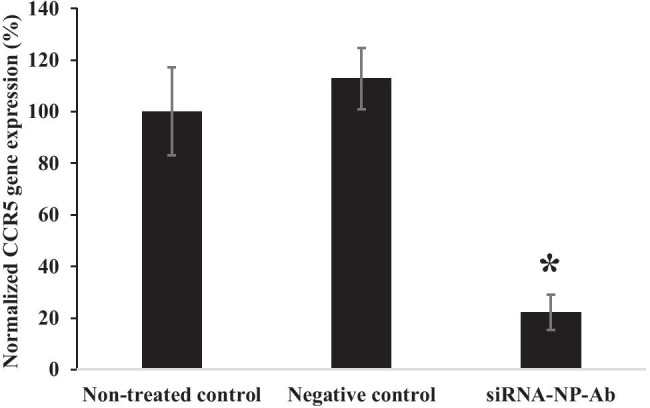
CCR5 gene knockdown in Sup-T1 cells incubated with IVR segments coated with pH-responsive Eudragit L100/PEG containing siRNA-NP-Ab (~ 19.09 µg of anti-CCR5 siRNA). Blank: Sup-T1 cells alone; negative control: consists of the IVR segment coated with the pH-responsive polymer but with drug-free SLNs. Data represents mean ± S.D.; *N* = 3. *P < 0.05 versus negative control

To evaluate the gene knockdown efficiency of the SLN containing siRNA, siRNA-NP-Ab were incubated directly with SupT-1 cells. Cells treated with 1.4 mg of siRNA-NP-Ab for 48 h (containing approximately 5 µg of siRNA) were able to achieve close to 60% CCR5 gene knockdown in 96-well plate (Fig. 5). IVR segments coated with 7 mg of siRNA-NP-Ab (containing around 2.84 µg of siRNA per mg of SLN) were able to release enough SLNs to significantly reduce CCR5 gene expression by 83 ± 5.1% in comparison to controls after 48 h of incubation in Sup-T1 cells in a 24-well plate (Fig. 6).

## Discussion

HIV continues to be a major global health concern particularly for women, who are disproportionally impacted due to biological and socio-cultural factors. Many of the current therapies fail due to low patient compliance and the development of drug resistance [39]. An IVR system that can provide prolonged drug release and is easily administered without the assistance of a healthcare practitioner will increase patient adherence [40]. Furthermore, using two different strategies that prevent HIV access to its target cells will greatly reduce infectivity and the potential to develop drug resistance. Our system is designed to deliver HCQ continuously for over 3 weeks to induce a T cell immune quiescent state within the FGT as the first-line of defense. During heterosexual intercourse, the second half of the IVR will rapidly release anti-CCR5 siRNA-NP-Ab to reduce the expression of CCR5 as the second-line of defense. However, further evaluation of our IVR system is required to determine whether or not drug resistance will develop.

HIV-exposed seronegative sex workers exhibit a low baseline of immune activation within the FGT [21]. It is believed that this immune quiescent state plays an important role in protecting against HIV infection. We decided to induce T cell immune quiescence using the immunomodulatory drug HCQ. It has been shown that HIV-exposed seronegative people exhibit low baseline immune activation with reduced expression of HLA-DR, CD38, CD69, and CCR5 on T cells [20, 41, 42]. In our study, HCQ was able to cross the hydrophilic PU wall of the IVR segments while immersed in buffer. The PU HP-60D-35 has a water swellability of 35% of its mass. The influx of water inside the polymer will aid in the diffusion of HCQ. To further control the diffusion of HCQ, a rate-controlling excipient HPMC was used. The concentration needed to induce immune quiescence in vitro is ~ 4.3 µg/mL [22, 43]. The IVR segment is capable of releasing at least 7 folds more than the required concentration to induce immune quiescence. We demonstrated in previous studies that HCQ released from HP-60D-35 was safe in a rabbit model, in the presence of vaginal (VK2/E6E7) and cervical (ECT-1/E6E7) epithelial cells, and in the presence of normal flora *Lactobacillus jensenii* and *crispatus* [32, 44]. The concentration of HCQ released in this study is significantly lower than the concentration reported that can induce cytotoxicity in vaginal cells (433.96 µg/mL) and *Lactobacillus jensenii and crispatus* (7 mg/mL) [32].

The second half of the IVR segment was fabricated to release anti-CCR5 siRNA-NP-Ab at high pH. The anti-CCR5 siRNA was mixed with PEI prior to encapsulation into SLNs. We believe once inside the cells, the siRNA can escape endosomal degradation via the proton sponge effect imparted by the positively charged PEI [45]. PEI has been shown to aid in the condensation of siRNA improving its encapsulation efficiency and assisting in the proton sponge effect by rupturing endosomes due to osmotic swelling.

To avoid unnecessary gene knockdown when it is not required, well-timed release of SLN is important. Release of SLNs can be triggered during sexual intercourse by the presence of seminal fluid inside the FGT. This can be achieved by using Eudragit L100 that will react with the change in pH environment due to the presence of seminal fluid. Eudragit L100 made of poly(methacrylic acid-co-methacrylate) is protonated and uncharged at acidic pH. At basic pH, it will become deprotonated and the negative charges repel themselves causing the polymer to swell [46, 47]. The swelling of the polymer leads to its disintegration and the release of the SLNs. For the fabrication of the pH-responsive film, we evaluated different solvents capable of dissolving Eudragit L100 (Table 1). The aim of this experiment was to find the most suitable solvent capable of dissolving only Eudragit L100 but not the PU matrix that it is coated on. Isopropanol appeared to completely dissolve Eudragit L100 and not have any visible effects on the PU HP-60D-35. Disintegration time of the pH-responsive film is also important since the rate of SLN release needs to be rapid during sexual intercourse. Since HCQ is continuously released to maintain T cell immune quiescence, it is assumed that this will provide sufficient time for the SLNs to be released, enter CD4^+^ T cells and silence CCR5 gene expression to prevent infection. In a non-human primate study, the authors have shown that sufficient infection was established only after 4 days [48]. Studies have shown that cervicovaginal mucus within the FGT may hinder nanoparticles from reaching the underlying submucosa [49]. As a result, the SLNs we prepared were coated with PEG that will potentially enhance the mucus penetration ability of the particles as described by others [50]. Assuming there is no mechanical tearing of the vaginal epithelium during sexual intercourse, we expect our SLNs to enter the target cells and knockdown CCR5 gene expression in less than 4 days. For the SLN release studies, C6, a hydrophobic fluorescent dye was used to monitor the SLNs. As expected, in VFS alone, very low amounts of SLN were released. To simulate sexual intercourse, 1 mL of SFS was added to the VFS. In a study by Fox et al., the authors measured changes in vaginal pH during sexual intercourse using radio-telemetry and found that low volumes of semen (1.5 mL) were capable of raising vaginal pH from 3.5 to 5.5 within 15 s [51]. At higher volumes of semen (5 to 6 mL), the vaginal pH can be raised from 4.3 to 7.2 in 8 s [51]. For our studies, we used 1 mL of SFS which was capable of raising the pH of VFS from 4.2 to 7.3. This volume was selected to mimic the lower range of ejaculated seminal fluid, which was sufficient in removing almost the entire pH responsive coating on the IVR segment. Human seminal fluid has a high buffering capacity and can easily change the vaginal tract pH from acidic to neutral and basic [52, 53].

In our studies, we determined that as low as 5 µg of siRNA was capable of knocking down gene expression by around 50%. This study also confirmed that there was sufficient uptake of SLN by Sup-T1 cells to significantly reduce CCR5 mRNA expression. It took 48 h for the siRNA to be released from the SLNs to induce gene knockdown. However, further studies must be performed in vivo to evaluate how fast the SLNs will reach the submucosa and to determine its efficiency in preventing HIV infection [48]. For in vivo studies, it is possible that other surrounding cells, e.g., vaginal epithelial cells, may uptake the SLN, as a result, functionalizing the SLN with anti-CD4 antibody will improve targeted delivery to CD4^+^ T cells. Lastly, we determined that the coated IVR segments were non-cytotoxic to VK2/E6E7 vaginal epithelial cells. We have previously shown that PU HP-60D-35 is stable under accelerated stressed conditions of 40 °C and 75% relative humidity [15], but further studies are needed to confirm the stability of the pH-responsive coating under the same conditions. Although room temperature may not affect the coating, it is unknown what impact countries with elevated climates and humidity may have.

## Conclusion

To the best of our knowledge, this is the first study to combine the delivery of HCQ and gene therapy as a novel strategy for preventing HIV infection. We were able to deliver HCQ continuously for over 3 weeks and also rapidly deliver siRNA-NP-Ab only at elevated pH environments such as those simulating the presence of seminal fluid. We were able to achieve significant CCR5 gene knockdown in comparison to controls, and the IVR segment was non-cytotoxic towards vaginal epithelial cells. Overall, combining chemotherapy and gene therapy into a single IVR is a promising platform that should be further explored for its potential as a microbicide.

## Data Availability

All data generated or analyzed during this study are included in this published article.
